# Hospital and Institutional News

**Published:** 1917-01-06

**Authors:** 


### HOSPITAL AND INSTITUTIONAL NEWS.

### THE CANADIAN MEDICAL SERVICE.

WITH praiseworthy despatch and promptitude the Special Board of Inquiry appointed by the Canadian Government to review the working of the Canadian Medical Service has been completed and presented. At the moment of going to press only a summary of its findings is available; but it is quite clear that the Special Board, which consisted of several senior Canadian officers, under the presidency of Sir William Babtie, V.C., of the British Army Medical Department, has supported Surgeon-General Carleton Jones, and has severely condemned both Sir Sam Hughes (the late Minister of Militia in Canada) and Inspector-General Bruce, of Toronto: it will be remembered that the latter reported very adversely upon Surgeon-General Jones' administration, and was ordered by Sir Sam Hughes to supplant him as Inspector-General in London. It is true that some of Colonel Bruce's minor recommendations are supported in the new report; but on the main points the Board pronounces emphatic disagreement from him. General Jones is especially commended for his zeal and industry and for the tactful performance of many delicate duties that fell to his lot. Colonel Bruce is charged with "ignoring the good work done by Surgeon-General Jones and his staff in circumstances of novelty and great difficulty," and with lack of intimate knowledge of Army organisation.

### THE SEGREGATION SCHEME DENOUNCED.

Most important of all, the scheme for the segregation of the Canadian wounded, which was one of the main principles of Colonel Bruce's programme, is given short shrift: in our own columns at the time it was ventilated a very strong protest against this extraordinary scheme was entered, and it is satisfactory to realise that it is now quite defunct. The Board points out that there are about twenty thousand Canadian casualties in hospitals in the United Kingdom. The capital expenditure necessary to provide accommodation for these patients under the concentration scheme would be not less than a million dollars; and this, as THE HOSPITAL pointed out at the time, is only the least of the many drawbacks attending the proposal, which the Board characterises as impracticable, expensive, and unnecessary. Nor does the Board find any desire amongst the Canadians themselves to be segregated in Canadian hospitals. For reasons of high policy it urges that such segregation is most undesirable, and tends to undo the bond of brotherhood sealed in the face of the enemy. So, too, in regard to research and other matters medical. The Board is of opinion that "as long as the Canadian Expeditionary Force forms an integral part of the Imperial Army . . . and so long as Canadian troops continue to operate under the command of the Commander-in-Chief, British Expeditionary Force, it must continue to be impossible to discriminate in the medical arrangements of Canadian and British troops." It would be as reasonable for British stretcher-bearers to refuse to pick up Canadian wounded on the battlefield as for the separation of the two Medical Services into water-tight compartments on this side of the Channel.

### THE V.A.D. HOSPITALS JUSTIFIED.

THERE was one other matter in Colonel Bruce's report which caused much searching of heart: that was his wholesale condemnation of the V.A.D. hospitals as inefficient, expensive, and unsatisfactory. The Board does not agree with these criticisms, but rather commends the V.A.D. hospitals for the vast amount of fine work they have accomplished. "The investigations of this Board do not support these allegations of inefficiency. The standard of professional efficiency naturally varies; but there is no ground, even in the special reports made by Colonel Bruce's direction, for the grave indictment contained in his report, 'a good deal of the surgery is bad.' . . . In no case has the Board had reason to be other than satisfied with the nursing in these institutions. The comments made in Colonel Bruce's report on the V.A.D. hospitals have been widely resented, and this Board is of opinion that these strictures are unjustified and regrettable." The inevitable sequel to this report of the Special Board of Inquiry is that Sir George Perley, the new Minister of Militia, has cancelled Colonel Bruce's appointment. Sir Sam Hughes has already disappeared into an oblivion from which he will be very unwise ever to attempt to emerge; and apparently the same is likely to hold good of the Inspector-General he sent over here, whose report caused such a sensation and has now been definitely repudiated by the findings of the Inquiry conducted under Sir W. Babtie's presidency .

### AT THE CLOSE OF THE WAR.

LOOKING ahead, there emerges the situation which will exist for the medical profession at the close of the war. According to some of the prophets, this situation is be a very unhappy one. Crowds of doctors returning to civil practice are to find themselves without occupation, and compelled, therefore, to accept any kind of work which will give them a livelihood. The supposition is that the practices of men who have been at the Front will have been monopolised by those who have remained at home. It is a poor compliment to the public to believe that patriotism and loyalty are at so low an ebb as is here suggested. To follow some writers in the press it might be supposed that doctors possess the power to retain patients against their will, and that no person can return to his usual adviser who has been away on military duty, because in the meantime other assistance has been necessary. The public knows this to be nonsense, and we doubt not that in most instances the returning practitioner will be welcomed, as he deserves to be, both by his colleagues and the community. The alarmist anticipations indulged in by some writers are without doubt exaggerated, and we are sure that where consideration and help are needed they will be supplied. Out of these troubles and disturbances there may be expected a greater development of corporate spirit in the profession, and the application of this may well lead to schemes of work which will bring advantage both to the individual patient and to the whole science and art of medicine.

### WANTED, A HOUSE AND GROUNDS, IN OR NEAR LONDON, FOR THE RED CROSS.

SIR JOHN COLLIE, the Medical Examiner to the London County Council and a great authority on neurasthenia, hysteria, and malingering, recently published in the *Times* a powerful plea for further treatment of soldiers discharged from the Army because of neurasthenia or other forms of nervous breakdown. The Joint War Committee of the British Red Cross Society and Order of St. John is prepared to provide the funds necessary for the equipment and maintenance of a suitable institution for the discharged neurasthenic soldier. Sir John Collie has been asked to take charge of the arrangements and has consented to do so. An endeavour is being made to obtain a large house or building with grounds, either in London or the immediate neighbourhood, sufficient to accommodate at least a hundred patients. Up to the end of the year these efforts had not met with success; and the Joint War Committee is therefore asking for offers of a suitable house or building, which should be addressed to the Hon. Arthur Stanley, chairman of the committee, British Red Cross Society, 83 Pall Mall, S.W.

### SOME OUTSTANDING BEQUESTS OF THE WEEK.

AT the New Year meeting of the governors of the Glasgow Royal Infirmary it was announced that the hospital will receive a legacy of £100,000 under the will of the late Miss Schaw, and it was also stated that other Glasgow hospitals will receive large sums from the same testatrix, whose estate is proved at close upon half a million sterling. Besides this enormous bequest those of Mr. W. H. Mudford, late editor and manager of the *Standard*, seem comparatively modest, though they are in fact by no means small. This gentleman left estate of the value of £65,000; the net personalty is about £55,000. After various specific bequests, which total about £17,000, three-quarters of the residue is left to be divided between five charities, two of which are hospitals. These two are the Middlesex Hospital (for the Cancer Charity) and the New Hospital for Women, Euston Road; roughly speaking, these institutions are entitled to £5,000 or £6,000 apiece.

### TWO MEDICAL VETERANS OF DISTINCTION.

WITHIN the past few days the death has occurred of two aged members of the medical profession, each of whom had a record of good work for London institutions in days gone by. On December 27 Richard Barwell died at Norwich, aged ninety years. He was consulting surgeon to Charing Cross Hospital and the author of several treatises on selected surgical subjects, chiefly connected with the joints and with deformities. Educated at St. Thomas's Hospital, he became a Member of the Royal College of Surgeons as far back as 1848, and took the Fellowship four years later. Mr. Barwell must, indeed, have been very near the top of the roll of the Fellows of that Society. Dr. William Cayley, who died on December 17, aged eighty, was a Fellow of King's College, London, and of the Royal College of Physicians. He was consulting physician to the Middlesex Hospital, to the Queen's Hospital for Children, and to the London Fever Hospital. He had been Senior Censor of the Royal College of Physicians, and contributed to medical literature on the subject of typhoid fever as recently as ten or twelve years ago.

### NATIONAL RELIEF GRANTS TO HOSPITALS.

THE principle of making grants to hospitals has been accepted by those responsible for the distribution of the Prince of Wales's Relief Fund, but, according to a recent return, distribution has not been carried beyond hospitals for soldiers. Lord Knutsford's Special Hospital for Officers has been awarded £10,000, and Queen Mary's Hospital for Limbless Soldiers and Sailors a like sum, while two other special hospitals for disabled soldiers have received £5,000 each. Of course the bulk of the money has been allotted to help associations such as the Soldiers' and Sailors' Families Association, which has received altogether from the Fund £2,291,088. These large grants are no doubt for current disbursement, and we welcome a gift to St. Dunstan's Hostel for Blinded Soldiers and Sailors of £35,000, which is wise and excellent indeed. There is no indication as yet of help to the voluntary hospitals, whether they are caring for wounded soldiers or not, but half a million while it is needed, carefully distributed throughout the country to these institutions, would help to cover the objects of the Prince of Wales's Fund quite as much as do most of the grants already announced.

### THE HYGIENE OF PRISONERS' CAMPS.

A SCHEME is reported by which the proper segregation of prisoners suffering from infectious diseases and the ascertainment and examination of complaints from prisoners could be secured through the aid of neutrals. At the recent Red Cross Conference, the conclusions of which have been summarised by the Stockholm correspondent of the *Morning Post*, it was suggested that medical commissions should be created, which would include a neutral surgeon, a medical officer of standing, and a Red Cross representative of the country to which the commission belongs. Such commissions should receive and examine complaints made by prisoners. But for certain appalling revelations we should not have thought that the segregation of infectious cases among prisoners required special neutral or Red Cross organisation. The creation of an independent channel for the examination of prisoners' complaints would no doubt be valuable. But where hygiene is neglected, as it has been by our enemies, can we hope that impartial examination would be encouraged? Red Cross work of the kind is always uphill work, however, and it is only up the Hill Difficulty that progress can be made.

### A REMARKABLE HOSPITAL CALENDAR.

The institutional calendar and Christmas or New Year card seems to have gone the way of many parochial calendars into unpopularity. Probably everyone remembers the large and forbidding sheet, resembling a blackboard covered with "sums," which in boyhood or girlhood made its appearance at this season. Too big to handle, with letters and figures too small to read conveniently, it was hung up somewhere, easily torn, and readily forgotten. To-day it is generally unregretted. But a hospital calendar need be none of these things, and to give readers an idea of how an institutional calendar may be designed free from these defects we draw attention to one which has been issued with deserved success by the Hampstead War Hospital Supply Depôt. Its attraction consists of a study of one of the members of the depôt at work on bandages, followed by a quotation for every day in the year. Each quotation has been chosen by a different worker, whose name appears with that of the author of the aphorism. The design on the calendar was conceived and carried out by Mr. T. Dudley Forsyth, whose wife is one of the workers in the depôt. The idea, which is capable of local variation in a hundred suggestive ways, is praiseworthy, and we commend it to those who do not forget the value of a hospital Christmas card. It was lately on sale at a function held in aid of the depôt.

### A BASE HOSPITAL AT STAINCLIFFE INSTITUTION?

The chance of establishing a base hospital at Dewsbury has entered on a new phase. The Guardians, after receiving a report of the proceedings of a recent deputation to Sir Alfred Keogh and to the Local Government Board, came to the conclusion that 350 men might be accommodated in the present building, and that a further 200 men might be provided for in temporary buildings on a site adjoining the infirmary and the nurses' home. The cost of the necessary additions will be £6,000, and the success of the scheme depends upon the raising of this sum by voluntary effort. Hope is entertained that the scheme may be realised by the end of March.

### BARGAINING FOR HOSPITAL TICKETS.

In a speech at the recent meeting of the general committee of the Birmingham Hospital Sunday Fund, Mr. F. Impey, an honorary secretary of the Fund, denounced the attempt of certain congregations to bargain for hospital tickets by refusing to hand over their collections free of conditions. He said that if such a practice extended the principle of the Sunday Fund collections would be imperilled, but that now the fact was known he hoped that those in control of the collections would prevent a bad example from spreading. Such a warning should remind all contributors that the Sunday Fund is a charity, and that its collections differ from "donations" on the one hand as much as they differ from workmen's weekly contributions on the other.

### THE LATE MR. T. FENWICK HARRISON.

Hospitals have lost a generous benefactor by the death on December 29 of Mr. Thomas Fenwick Harrison. Mr. Harrison was a member of a wealthy Liverpool shipping family, but at the age of thirty-seven he retired from business and devoted himself to the life of a country gentleman, having a lovely seat at King's Walden, in Hertfordshire. This fine place he turned into a private hospital to accommodate sixty wounded soldiers, and his daughter took charge of it. Mr. Harrison is perhaps best remembered as the gentleman who gave £6,000 for the Kitchener letter at the Red Cross sale. Among his benefactions last year were £1,000 to St. Bartholomew's Hospital, and a number of motor-ambulances to the Red Cross Society. It was his custom to motor up to London from Hitchin several times a week with a car full of fruit and flowers for the patients at the Army hospital at Millbank, and the institution kept by "Sister Agnes" in Grosvenor Gardens.

### THE WELSH MEDICAL SCHOOL.

It is officially intimated that there is now every indication that Wales will soon have its complete National Medical School, the obstacles placed in the way of the project by Government Departments having been overcome. The scheme, already outlined in The Hospital, is framed on ambitious lines. The buildings are provided for by Sir William James Thomas's fine gift of £100,000; and it has been decided, in order to place the school in the very front rank, that the professors of medicine, surgery, midwifery, etc., shall be whole-time men, who will devote themselves wholly to teaching and their hospital work. The endowment of the several Chairs is expected from the Treasury. The one thing now needed in order to complete the scheme is the endowment of scholarships, exhibitions, etc., and an appeal is being made to the generous public on the students' behalf in these respects. Mrs. John Nixon, whose name is already prominently associated with the undertaking, has led the way by giving a sum sufficient to endow a research scholarship in medicine and medical pathology—namely, £2,500; and Lord Merthyr has endowed with a similar sum a research scholarship in cancer. All the scholarships and medals, it is stated, will be open to women students. Notwithstanding the war and the fact that fifty students went into the Army last year, there are at present in the school attached to the University College of South Wales and Monmouthshire over sixty medical students. The promoters hope to be able to attract between 300 and 400 students and to develop the school into one of the largest in the country.

### CONTROL OF WAR CHARITIES.

The Charity Commissioners, in continuing their work of regulation and co-ordination, have, in view of the establishment of the Central Prisoners of War Committee, made arrangements for the handing over to that Committee of the balance of the British Prisoners of War in Germany Fund, formerly conducted under the supervision of a weekly journal. In respect of other charities with similar objects the Commissioners have allowed the funds to be wound up under the supervision of the executive in each case without taking any further steps within their powers, and still two further prisoners' charities are under supervision. In view of the multiplicity of war charities generally, this vigorous method of amalgamation and prevention of overlapping will be welcomed. The activities of the Commissioners have extended to thirteen charities under Section 7 of the War Charities Act, and, in the case of five of them, schemes have been established for their regulation. The affairs of the Belgian Soldiers Fund has been before the Commissioners, but, after explanations and evidence had been given by the present administrators, the winding-up of the Fund was left in their hands.

### A STEP RETRACED.

The Swansea Health Committee has decided to refer back the minute which declared that "no further payment be made to the Welsh National Memorial Association until a copy of the Association's balance-sheet, as audited by the Treasury auditor, has been supplied to the Corporation." It was stated that the Director of the Welsh National Memorial Association had visited Swansea and removed any unsatisfactory impression from the borough treasurer's mind. The matter was discussed in The Hospital on December 23 last.

### LONDON HOSPITAL SATURDAY RESULTS.

The executive of the Hospital Saturday Fund and its large organisation of local secretaries and collectors must have been faced with many difficult problems during the past year. Employment has, of course, been good; but the effect of the constant changes brought about by helpers being called up for military service, or taking up different work of greater national importance in another locality, has meant that the machinery of collection of funds has been in a constant state of dislocation. It says a great deal for the loyalty and energy of all the workers that the net result for 1916, as calculated up to December 9, is appreciably an advance on the corresponding period in 1915, the receipts being respectively £24,331 and £31,852. At the last meeting of the board of delegates, when this increase was referred to, the Chairman, Sir T. Vezey Strong, said that very liberal contributions had been received from munition workers, and that the Ambulance Committee were alive to the importance of supplying the centres where these helpers were employed with ample first-aid requisites. We are glad to hear good accounts have been received of all members of the Head Office Staff on active service.

### A SUGGESTION FOR THE BOARD OF THE KING EDWARD VII. HOSPITAL, CARDIFF.

The Welsh Housing and Development Association have forwarded a suggestion to the board of management of the King Edward VII. Hospital that further extensions of the institution shall be carried out on one of the elevated regions in the suburban areas of Cardiff, rather than in the confined district of Newport Road, Cardiff, where the hospital is situated.

### THE FUNCTION OF SANATORIA.

The confusion in the public mind concerning the place of the sanatorium in the anti-tuberculosis campaign has never recovered from the politician's reckless allusion to "first-class hotels." Every opportunity, therefore, must be seized to state the truth, that it be not put out of countenance by the ceaseless iteration of similar falsehoods. Dr. Marcus Paterson, medical director of the Welsh National Memorial Association, in the report for the year ended on March 31, 1916, again forcibly reminds the public that the sanatoria "will not of themselves eradicate tuberculosis," that "sanatorium treatment is only one method" among "the combination of all known methods" required "to combat this disease." Institutional accommodation for advanced cases is the most neglected need of the moment. Why this is grudged we cannot make out. There is no better way of preventing the spread of the disease.

### THIS WEEK'S DRUG MARKET.

Business has hardly commenced in real earnest, but nevertheless there is more activity than is usually the case so early in a New Year. In synthetic drugs there are few changes in value, but the tendency in aspirin, salicylic acid, and salicylate of soda is still towards lower prices. On the other hand, phenacetin continues very scarce; supplies, however, are expected to arrive from the United States shortly, when we may see some reduction in price. Phenazone is rather dearer. Acetic acid is again moving upwards in price. Chamomiles are extremely scarce. The price tendency of castor oil is still in an upward direction. Quinine is quiet, but holders show little inclination to make concessions. A revision of the prices of spirit is expected to take place, and it need hardly be said that such revision, if it comes to pass, will not be downwards. Cantharides are dearer. Oil of wintergreen is rather lower in price.

### TO OUR READERS.

**Contributions are specially invited from any of our readers to these columns. They should deal with topical subjects and news. They must be authenticated for the information of the Editor only. The minimum payment if published is 5s. There is no hard-and-fast rule as to space, but notes of about twenty lines in length are preferred.**

